# Neurokinin-1 Receptor Antagonist Treatment in Polymicrobial Sepsis: Molecular Insights

**DOI:** 10.4061/2010/601098

**Published:** 2010-09-20

**Authors:** Akhil Hegde, Yung-Hua Koh, Shabbir M. Moochhala, Madhav Bhatia

**Affiliations:** ^1^Cardiovascular Biology Program, Department of Pharmacology, Yong Loo Lin School of Medicine, National University of Singapore, MD 11, No. 05-09, 10 Medical Drive, Singapore 117597; ^2^Department of Pathology, University of Otago, Christchurch, 2 Riccarton Avenue, P.O. Box 4345, Christchurch 8140, New Zealand; ^3^DSO National Laboratories, DSO@DMERI, Kent Ridge, 27 Medical Drive, Singapore 117510

## Abstract

Neurokinin-1 receptor blocking has been shown to be beneficial against lung injury in polymicrobial sepsis. In this paper, we evaluated the possible mediators and the mechanism involved. Mice were subjected to cecal ligation and puncture (CLP-) induced sepsis or sham surgery. Vehicle or SR140333 [1 mg/kg; subcutaneous (s.c.)] was administered to septic mice either 30 min before or 1 h after the surgery. Lung tissue was collected 8 h after surgery and further analyzed. CLP alone caused a significant increase in the activation of the transcription factors, protein kinase C-*α*, extracellular signal regulated kinases, neurokinin receptors, and substance P levels in lung when compared to sham-operated mice. SR140333 injected pre- and post surgery significantly attenuated the activation of transcription factors and protein kinase C-*α* and the plasma levels of substance P compared to CLP-operated mice injected with the vehicle. In addition, GR159897 (0.12 mg/kg; s.c.), a neurokinin-2 receptor antagonist, failed to show beneficial effects. We conclude that substance P acting via neurokinin-1 receptor in sepsis initiated signaling cascade mediated mainly by protein kinase C-*α*, led to NF-*κ*B and activator protein-1 activation, and further modulated proinflammatory mediators.

## 1. Introduction

Sepsis is an intense systemic inflammatory response syndrome (SIRS) generally caused by bacterial infection [1]. Substance P (SP), a *preprotachykinin-A* (*PPTA*) gene product, is an immunoregulatory neuropeptide implicated in various inflammatory diseases including sepsis. We have previously shown that *PPTA *gene knock-out mice are protected significantly against polymicrobial sepsis [2], and neurokinin-1 receptor (NK-1R) antagonist treatment was beneficial against lung injury in mouse sepsis model [3]. The mechanism by which NK-1R blocking protects against lung injury was yet to be elucidated. 

The NF-*κ*B transcription factor system is known to control the expression of a number of genes involved in the innate immune response of the body against infection and inflammation. Genes responsible for immunoreceptors, cytokines, chemokines, and apoptosis are all modulated by this important family of transcription factors [4]. NF-*κ*B activity is reported to be impaired in chronic inflammation [5], and inhibition of NF-*κ*B has been suggested to be beneficial in maintaining the balance between pro- and anti-inflammatory cytokines [6]. Activator Protein-1 (AP-1) is another transcription factor that is induced by inflammatory cytokines and cellular stress. Phosphorylation of AP-1 is necessary for transcriptional activity.

Phosphorylation of NF-*κ*B and AP-1 and thus transcription of proinflammatory mediators are facilitated by the activation of various mitogen-activated protein kinases (MAPKs). MAPKs in turn are activated by bacterial products, cytokines, and chemokines [7, 8]. AP-1 c-Jun is reported to be phosphorylated *in vitro *by extracellular signal-regulated kinases (ERK1 and ERK2) [9, 10]. ERK is also shown to be a regulator of NF-*κ*B activity [11]. ERK1/2 reportedly induce NF-*κ*B activation by stimulating downstream MAPK-activated protein kinases [12, 13]. p38 MAPKs are also activated by inflammatory cytokines and environmental stress.

 We explored the possible downstream mediators and transcription factors involved in NK-1R antagonism in sepsis. Apart from analyzing the activation of NF-*κ*B and AP-1, protein levels of MAPKs, protein kinase C (PKC) isoforms, mRNA levels of NK-1R, neurokinin-2 receptor (NK-2R), and SP concentrations were also evaluated. Although the effects of SP were found to be mediated mainly via NK-1R in sepsis, it was interesting to explore if NK-2R had any role in the actions of SP in sepsis. Thus, we also studied the effect of blocking NK-2R with GR159897, a highly potent, selective, and long-acting nonpeptide NK-2R antagonist, in polymicrobial sepsis. Lung myeloperoxidase (MPO) activity, chemokine, and cytokine levels were measured to evaluate the beneficial effects, if any, of NK-2R antagonism in sepsis.

## 2. Materials and Methods

### 2.1. Animal Ethics

All animal experiments performed were in accordance with the guidelines of the DSO Animal Care and Use Committee (DSOACUC), Singapore, which follows the established International Guiding Principles for Animal Research. Mice were maintained at a controlled temperature (21–24°C) and lighting (12 hours light/dark cycles) and fed with standard laboratory chow and drinking water, provided *ad libitum*.

### 2.2. Induction of Polymicrobial Sepsis

Swiss mice (male, 25–30 g) used for the study were randomly assigned to sham or cecal ligation and puncture (CLP) experimental groups (*n* > 6 in each group). Polymicrobial sepsis was induced in mice by CLP as described earlier [14–17]. The same surgical procedure except cecal ligation and puncture was performed on sham-operated animals. Vehicle (DMSO diluted in PBS, 0.25% v/v) or SR140333 [1 mg/kg; 0.25 mg/mL, subcutaneous (s.c.)] was administered to CLP-operated mice either 30 minutes before (pretreatment) or 1 hour after (posttreatment) the CLP. 

 Another identical set of mice were subjected to either sham or CLP surgery as above, and the CLP group of mice were injected with vehicle (DMSO diluted in PBS, 0.25% v/v) or GR159897 (Tocris Bioscience, Missouri, USA) (0.12 mg/kg; 0.25 mg/mL, s.c.) 1 hour after CLP. GR159897 is reported to be highly potent and specific in antagonizing NK-2R with affinity in subnanomolar range [18]. GR159897 (0.12 mg/kg; i.v.) has been shown to antagonize bronchoconstriction induced by NK-2R agonist (28 times) in guinea-pig and also negligibly affect NK-1R and NK-3R [19]. Thus, we chose a small dose (0.12 mg/kg, s.c.) of GR159897 to be sufficient to block NK-2R.

The animals were sacrificed 8 hours after surgery by an i.p. injection of a lethal dose of pentobarbitone (Jurox Pty Ltd, Rutherford, NSW, Australia). Blood was collected by cardiac puncture, heparinized, and centrifuged, and plasma was removed and stored at −80°C. Samples of lung were snap frozen in liquid nitrogen and stored at −80°C for subsequent measurement.

### 2.3. Preparation of Nuclear Extract

Nuclear extracts were prepared from lung tissue using Active Motif nuclear extraction kit (Carlsbad, CA, USA) following the instructions from the manufacturer. Briefly, lung tissue (50 mg) was homogenized in hypotonic buffer containing detergent, incubated for 15 minutes on ice, and then centrifuged at 850 g, 4°C for 10 minutes. The pellets were resuspended in hypotonic buffer, treated with detergent, and centrifuged at 14,000 g, 4°C for 30 seconds. The nuclei in the pellets were lysed with complete lysis buffer and the nuclear proteins solubilized in the buffer containing protease inhibitors. The nuclear fraction was separated by centrifuging at 14,000 g, 4°C for 10 minutes and collecting the supernatant. Protein concentration in the nuclear extract was determined by using Bradford protein assay kit (Bio-Rad Laboratories, CA, USA). Protein concentration was calculated using a standard curve.

### 2.4. NF-*κ*B DNA-Binding Activity

ELISA-based TransAM NF-*κ*B p65 transcription factor assay kit (Active Motif, Carlsbad, CA, USA) was used to measure NF-*κ*B binding to DNA and activation, as per the manufacturer's instruction. Nuclear proteins (5 *μ*g) from the nuclear extract were added to each well coated with an unlabeled oligonucleotide containing the consensus binding site for NF-*κ*B (5′-GGGACTTTCC-3′) [20] and incubated for 1 hour at room temperature to allow the active form of NF-*κ*B to bind. A primary antibody directed against activated NF-*κ*B p65 subunit was added to detect the NF-*κ*B complex bound to the oligonucleotide. Addition of a secondary antibody conjugated to horseradish peroxidase (HRP) provided a sensitive colorimetric estimation by spectrophotometry. Absorbance was measured at 450 nm using microplate reader (Tecan Systems Inc., San Jose, CA, USA).

### 2.5. AP-1 DNA-Binding Activity

TransAM AP-1 c-Jun transcription factor assay kits (Active Motif, Carlsbad, CA, USA) were used to detect and quantify AP-1 activation. AP-1 dimers in the nuclear extract (5 *μ*g of protein) were added to the 96-well microplate with immobilized oligonucleotide that had a 12-O-tetradecanoyl-phorbol-13-acetate (TPA)-responsive element (TRE) (5′-TGA(C/G)TCA-3′) to specifically bind to the oligonucleotide. Primary antibody was used to recognize accessible epitopes on c-Jun proteins upon DNA binding. Secondary antibody conjugated to HRP was added for the colorimetric reaction. Absorbance was read at 450 nm using microplate reader (Tecan Systems Inc., San Jose, CA, USA).

### 2.6. Western Blot Experiment

Protein levels of I*κ*B*α*, PKC*α*, PKC*δ*, PKC*ε*, and MAPKs in lung homogenates were analyzed by Western blot. 80 *μ*g of the lung protein was separated on a 12% SDS-polyacrylamide gel (Invitrogen, Carlsbad, CA, USA) and transferred to PVDF membranes (Millipore, MA, USA) by electrophoresis. Nonspecific binding was blocked by incubating the membrane at room temperature in 5% nonfat dry milk in phosphate-buffered saline Tween 20 (PBST) (0.05% Tween 20 in phosphate-buffered saline) for 1 hour. The blots were incubated overnight at 4°C with primary antibody (Cell Signalling Technology) at 1 : 1000 dilutions in 2.5% nonfat dry milk in PBST. The membranes were then washed four times with PBST and incubated with goat anti-rabbit HRP-conjugated secondary antibody (Santa Cruz Biotechnology) at 1 : 2000 dilutions in 2.5% nonfat dry milk in PBST for 2 hours. Visualization of the blot was done using enhanced chemiluminescence (ECL) detection kit (Pierce, Rockford, IL, USA) and with exposure to X-ray films (CL-XPosure, Pierce). Hypoxanthine guanine phosphoribosyl transferase (HPRT) (Santa Cruz Biotechnology; 1 : 1000 dilution) was used as the housekeeping protein. The band densities were quantified using a UVP bioimaging system (UVP, Upland, CA, USA). The intensity of bands was analyzed using LabWorks Image Analysis software (UVP, CA, USA) and expressed as integrated optical density (IOD).

### 2.7. RNA Isolation and Quantification

Total RNA was isolated from the lung tissue (*n* > 6 for each group) using TRIzol reagent (Invitrogen, Carlsbad, CA, USA) according to the manufacturer's protocol. RNeasy mini kit was used to clean up the total RNA after extraction. Briefly, extracted RNA sample was lysed and homogenized in the presence of a highly denaturing guanidine-thiocyanate-containing buffer to inactivate RNases leaving intact RNA. Ethanol was added for appropriate binding, and the sample was applied to an RNeasy Mini spin column to bind total RNA to the membrane. Contaminants were washed away and high-quality RNA was eluted in 30–100 *μ*L water. The quantity of extracted RNA was determined by spectrophotometric analysis (NanoDrop ND1000). RNA samples with A_260_/A_280_ ratios close to 2.0 (range: 1.9–2.1) and integrity were used for reverse transcription-polymerase chain reaction (RT-PCR). The integrity of RNA was assessed by 1% w/v denaturing agarose gel electrophoresis using GelRed dye to stain 18S and 28S rRNA bands. The RNA sample was stored at −80°C until RT-PCR.

### 2.8. Semiquantitative RT-PCR

Isolated lung RNA (1 *μ*g) was reversely transcribed using iScript cDNA Synthesis Kit (Bio-Rad, Hercules, CA, USA) at 25°C for 5 minutes, 42°C for 30 minutes, followed by 85°C for 5 minutes. The cDNA was used as a template for PCR amplification by iQ Supermix (Bio-Rad, Hercules, CA, USA). The primer sequences and optimal amplification conditions for NK-1R, NK-2R and 18S gene are given in Table 1. PCR amplification was carried out in MyCycler (Bio-Rad). The reaction mixture was first subjected to 95°C for 3–5 minutes, followed by an optimal cycle of amplification and a final extension at 72°C for 5–7 minutes. PCR products were analyzed on 1.5% w/v agarose gel containing 0.1 *μ*L/mL GelRed and visualized by the UVP bioimaging system (UVP, Upland, CA, USA). The intensity of bands was analyzed using LabWorks Image Analysis software (UVP). Densitometry results (IOD) from PCR products were normalized to the mouse 18S band densities.

### 2.9. Substance P Estimation

SP levels were measured in lung and plasma using competitive ELISA kit (Bachem, Peninsula Laboratories, USA) as per the manufacturer's protocol. Briefly, the lung tissue was homogenized in 1 mL ice-cold SP assay buffer for 20 seconds. The homogenate was centrifuged (13 000 rpm, 20 minutes, 4°C), and the supernatant was separated. SP in the supernatant was adsorbed on C18 separation column containing 200 mg C18 (Bachem, Peninsula Laboratories, USA), as described in [21]. The adsorbed peptide was then eluted with 1.5 mL of 75% v/v acetonitrile and freeze dried overnight. The lyophilized sample was reconstituted in SP assay buffer, and the absorbance was measured at 450 nm. SP level was read from a standard curve and expressed as nanograms per milliliter for plasma and picograms per microgram of DNA for lung (corrected for the DNA content of the tissue using Hoechst dye 33256 [22]).

### 2.10. Myeloperoxidase (MPO) Activity and ELISA Analysis

MPO activity, chemokine (CCL-2 and CXCL-2), and cytokine (IL-6 and IL-1*β*) levels in lung were quantified as described elsewhere [3]. Briefly, tissue samples were homogenized in 20 mM phosphate buffer (pH 7.4) and centrifuged (10 000 × g, 10 minutes, 4°C), and the resulting pellet was resuspended in 50 mM phosphate buffer (pH 6.0) containing 0.5% hexadecyltrimethylammonium bromide (Sigma, St. Louis, MO, USA). The suspension was subject to four cycles of freezing and thawing and further disrupted by sonication (40 seconds). The sample was then centrifuged (10 000 × g, 5 minutes, 4°C), and the supernatant was used for the MPO assay. The reaction mixture consisted of the supernatant, 1.6 mM tetramethylbenzidine (Sigma, St. Louis, MO, USA), 80 mM sodium phosphate buffer (pH 5.4), and 0.3 mM hydrogen peroxide. This mixture was incubated at 37°C for 110 seconds, the reaction was terminated with 2 M H_2_SO_4_, and the absorbance was measured at 450 nm. The absorbance was then corrected for the DNA content of the tissue sample [22].

For the ELISA analysis, anti-chemokine/cytokine primary antibody was coated onto 96-well ELISA plates and incubated overnight at room temperature. Samples and standards were added to the wells and incubated for 2 hours, the wells were washed, and a biotinylated goat anti-mouse chemokine/cytokine antibody was added for 2 hours. Plates were washed again, and streptavidin conjugated to HRP was added for 20 minutes. After a further wash, tetramethylbenzidine was added for color development, and the reaction was terminated with 2 N H_2_SO_4_. Absorbance was measured at 450 nm. Sample concentration was estimated from the standard curve. DNA assay was performed fluorometrically by using Hoechst dye 33256 [22]. The sample concentration was then corrected for the DNA content of the tissue [22].

### 2.11. Statistical Analysis

All values were expressed as mean ± S.E.M. The significance of changes was evaluated by using ANOVA when comparing three or more groups and Tukey's method as a post hoc test for comparison among different groups. A *P* value of <.05 was considered to indicate a significant difference.

## 3. Results

### 3.1. Effect of SR140333 Treatment on Lung NF-*κ*B Activation after Sepsis

**Figure 1:**
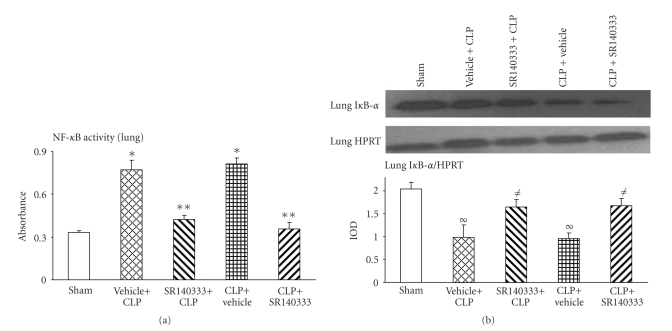
Effect of SR140333 administration, either 30 minutes before or 1 hour after CLP, on lung NF-*κ*B DNA-binding activity. Mice (*n* = 6–9 in each group) were divided into CLP-operated and sham-operated groups. CLP-operated mice received vehicle (DMSO in PBS, 0.25% v/v) or SR140333 (1 mg/kg; 0.25 mg/mL) s.c. either 30 minutes before (pretreatment) or 1 hour after (posttreatment) the CLP. Same surgical procedure as the CLP-operated animals except the cecal ligation and puncture was performed on sham-operated animals. 8 hours after the CLP procedure, mice were sacrificed, and lung (a) NF-*κ*B DNA-binding activity and (b) I*κ*B-*α* level (representative I*κ*B-*α* and HPRT control bands shown on the upper panel) were determined. Results shown are the mean ±S.E.M. “Vehicle + CLP” and “SR140333 + CLP” represent the groups that received vehicle and SR140333 treatment, respectively, commencing 30 minutes prior to CLP. “CLP + vehicle” and “CLP + SR140333” represent the groups that received vehicle and SR140333 treatment, respectively, 1 hour after CLP. *P < .001 when vehicle-treated CLP animals were compared with sham group animals; **P < .001 when SR140333-treated CLP animals were compared with vehicle-treated CLP animals; ^*∞*^ *P* < .01 when vehicle-treated CLP animals were compared with sham group animals; ^≠^ *P* < .05 when SR140333-treated CLP animals were compared with vehicle-treated CLP animals. CLP: cecal ligation and puncture; HPRT: Hypoxanthine guanine phosphoribosyl transferase; IOD: integrated optical density.

As NF-*κ*B is an important transcription factor involved in inflammatory diseases, activation and nuclear translocation of NF-*κ*B were measured after induction of sepsis and treatment with the NK-1R antagonist. NF-*κ*B activity was significantly increased (*P* < .001) in vehicle-treated (both pre- and posttreatment) mice 8 hours after CLP compared to the sham group (Figure 1(a)). Injection of SR140333, both 30 minutes before and 1 hour after CLP, reduced the NF-*κ*B activity significantly (*P* < .001) (Figure 1(a)).

Western blot analysis was performed to evaluate the activation and degradation of I*κ*B*α*. When the inhibitory protein I*κ*B*α* is phosphorylated and degraded, NF-*κ*B is freed for nuclear translocation. As expected, we observed a significant reduction in I*κ*B levels (*P* < .001) in vehicle-treated (both pre- and posttreatment) mice 8 hours after CLP compared to the sham group (Figure 1(b)). SR140333 treatment, both 30 minutes before and 1 hour after CLP, restored the I*κ*B levels significantly (*P* < .05) (Figure 1(b)). 

### 3.2. Effect of SR140333 Treatment on Lung AP-1 Activation after Sepsis

**Figure 2:**
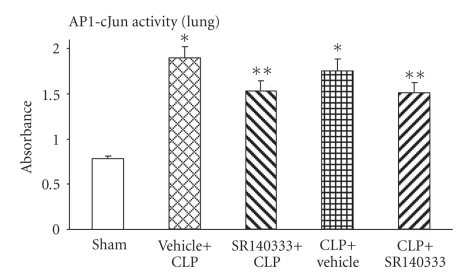
Effect of SR140333 administration, either 30 minutes before or 1 hour after CLP, on lung AP-1 activity. Results shown are the mean ±S.E.M (*n* = 8-9 in each group). “Vehicle + CLP” and “SR140333 + CLP” represent the groups that received vehicle and SR140333 treatment, respectively, commencing 30 minutes prior to CLP. “CLP + vehicle” and “CLP + SR140333” represent the groups that received vehicle and SR140333 treatment, respectively, 1 hour after CLP. *P < .001 when vehicle-treated CLP animals were compared with sham group animals; **P < .05 when SR140333-treated CLP animals were compared with vehicle-treated CLP animals. AP-1: activator protein-1; CLP: cecal ligation and puncture.

Activation of another transcription factor that is involved in sepsis, AP-1 c-Jun, was also measured after induction of sepsis and treatment with the NK-1R antagonist. 8 hours after CLP, AP-1 activity was significantly increased (*P* < .001) compared to the sham group in vehicle-treated mice (Figure 2). S.c administration of the NK-1R antagonist, SR140333, both 30 minutes before and 1 hour after CLP, reduced the AP-1 activity significantly (*P* < .001).

### 3.3. Effect of SR140333 Treatment on MAPKs and PKC Isoforms in Sepsis

**Figure 3:**
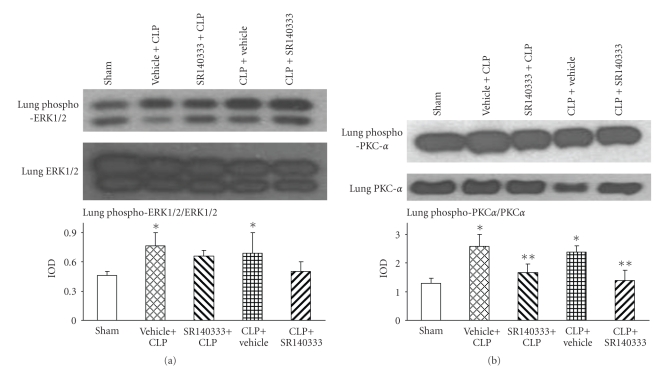
Effect of SR140333 administration, either 30 minutes before or 1 hour after CLP, on lung (a) Phospho ERK1/2 and (b) Phospho-PKC*α*. Results shown are the mean ±S.E.M (*n* = 6 in each group). “Vehicle + CLP” and “SR140333 + CLP” represent the groups that received vehicle and SR140333 treatment, respectively, commencing 30 minutes prior to CLP. “CLP + vehicle” and “CLP + SR140333” represent the groups that received vehicle and SR140333 treatment, respectively, 1 hour after CLP. Upper panels show representative western blot gel pictures of phosphorylated and total ERK1/2 and PKC*α* for each of the groups. *P < .05 when vehicle-treated CLP animals were compared with sham group animals; **P < .05 when SR140333-treated CLP animals were compared with vehicle-treated CLP animals. CLP: cecal ligation and puncture; ERK: extracellular signal regulated kinase; IOD: integrated optical density; PKC: protein kinase C.

To evaluate the link between NK-1R antagonist treatment and transcription factor inhibition, western blot analysis was performed for various MAPKs: ERK1/2, p38, and JNK. Significant activation of ERK1/2 to the phosphorylated form was detected 8 hours after CLP in vehicle-treated mice lung homogenates (Figure 3(a)). SR140333 treatment, both 30 minutes before and 1 hour after CLP, showed a trend to reduce the phospho ERK1/2 levels, although the reduction was not statistically significant (Figure 3(a)). p-p38 and p-JNK MAPKs showed very weak signals and did not show significant differences between the groups (data not shown).

The enzyme PKC involved in signal transduction of G protein-coupled receptors (GPCRs) was also evaluated in sepsis. Significant phosphorylation and activation of PKC*α* was observed 8 hours after sepsis in mice injected with only vehicle compared to the sham group (Figure 3(b)). Blocking of NK-1R with SR140333 (both pre- and posttreatment) resulted in a significant reduction in lung PKC*α* phosphorylation in mice 8 hours after sepsis induction. There were no statistically significant differences in PKC*δ* and PKC*ε* phosphorylation between groups (data not shown).

### 3.4. Effect of SR140333 Treatment on Lung NK Receptor Expression after Sepsis

**Figure 4:**
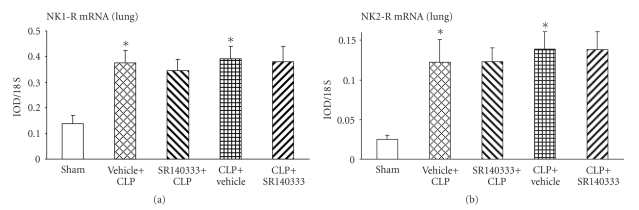
Effect of SR140333 administration, either 30 minutes before or 1 hour after CLP, on lung (a) NK-1R and (b) NK-2R mRNA levels. Results shown are the mean ± S.E.M (*n* = 6–9 in each group). “Vehicle + CLP” and “SR140333 + CLP” represent the groups that received vehicle and SR140333 treatment, respectively, commencing 30 minutes prior to CLP. “CLP + vehicle” and “CLP + SR140333” represent the groups that received vehicle and SR140333 treatment, respectively, 1 hour after CLP. *P < .05 when vehicle-treated CLP animals were compared with sham group animals. CLP: cecal ligation and puncture; IOD: integrated optical density; NK-1(2)R: neurokinin-1(2) receptor.

mRNA levels of NK receptors, NK-1R and NK-2R, were analyzed by semiquantitative RT-PCR. CLP-induced sepsis resulted in a significant upregulation of both the NK receptors in vehicle-treated mice compared to sham group (Figures 4(a) and 4(b)). NK-1R blocker had no significant effect on the expression of NK-1R and NK-2R. However, SR140333 treatment 30 minutes before CLP showed a slight, but nonsignificant, reduction in the expression of NK-1R.

### 3.5. Effect of SR140333 Treatment on SP Levels in Sepsis

**Figure 5:**
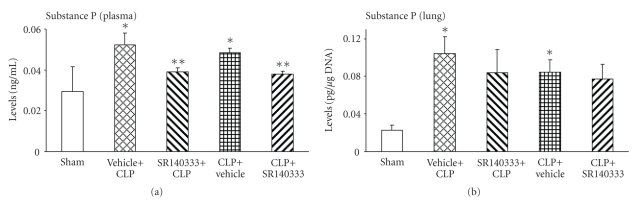
Effect of SR140333 administration, either 30 minutes before or 1 hour after CLP, on (a) plasma and (b) lung SP levels. Results shown are the mean ± S.E.M (*n* = 6–9 in each group). “Vehicle + CLP” and “SR140333 + CLP” represent the groups that received vehicle and SR140333 treatment, respectively, commencing 30 minutes prior to CLP. “CLP + vehicle” and “CLP + SR140333” represent the groups that received vehicle and SR140333 treatment, respectively, 1 hour after CLP. *P < .01 when vehicle-treated CLP animals were compared with sham group animals; **P < .05 when SR140333-treated CLP animals were compared with vehicle-treated CLP animals. CLP: cecal ligation and puncture; SP: substance P.

Next, we measured the SP levels in plasma and lung. Consistent with literature reports, systemic (Figure 5(a)) and lung tissue (Figure 5(b)) SP levels were elevated in mice subjected to CLP surgery. Treatment with SR140333 did not affect the lung SP levels (Figure 5(b)). However, plasma SP levels were significantly reduced by the NK-1R antagonist, injected either 30 minutes before or 1 hour after surgery (Figure 5(a)).

### 3.6. Effect of GR159897 Treatment on Neutrophil Sequestration, Chemokine, and Cytokine Levels in Lung after CLP Surgery

**Figure 6:**
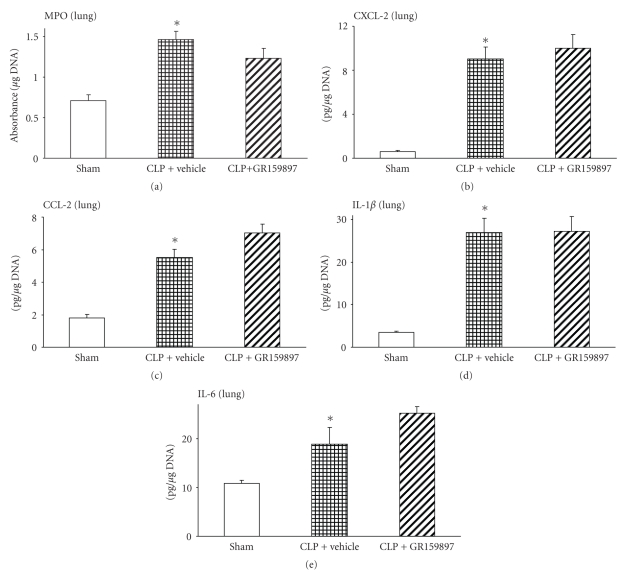
Effect of GR159897 administration 1 hour after CLP on lung (a) neutrophil infiltration (MPO), (b) CXCL-2, (c) CCL-2, (d) IL-1*β* and (e) IL-6 levels. Results shown are the mean ± S.E.M (*n* = 6–9 in each group). “CLP + vehicle” and “CLP + SR140333” represent the groups that received vehicle and SR140333 treatment, respectively, 1 hour after CLP. *P < .001 when vehicle-treated CLP animals were compared with sham group animals. CLP: cecal ligation and puncture; IL: interleukin; MPO: myeloperoxidase.

Lung MPO activity, a measure of neutrophil infiltration, was significantly increased in vehicle-treated animals when compared to the sham mice (Figure 6(a)) 8 hours after CLP. However, treatment with the NK-2R antagonist, GR159897, 1 hour after CLP, did not significantly reduce the MPO activity in lung (Figure 6(a)). Similarly, we measured the lung levels of major CXC chemokine, CXCL-2, and CC chemokine, CCL-2, and cytokines, IL-1*β* and IL-6, in lung homogenates. CLP-induced sepsis resulted in a significantly higher CXCL-2 level in vehicle-treated mice compared to the sham group (Figure 6(b)). However, GR159897 treatment did not change the elevated lung CXCL-2 levels observed 8 hours after CLP surgery in vehicle control group (Figure 6(b)). Further, CCL-2 levels also increased significantly 8 hours after CLP surgery without GR159897 administration compared to that of sham animals (Figure 6(c)), but this increase in CCL-2 levels was not affected significantly by GR159897 administration 1 hour after CLP surgery (Figure 6(c)).

 Animals injected only with the vehicle showed a significant increase in lung IL-1*β* (Figure 6(d)) and IL-6 (Figure 6(e)) levels 8 hours after CLP surgery compared to that in sham mice. Administration of GR159897 1 hour after CLP procedure failed to affect the lung IL-1*β* (Figure 6(d)) and IL-6 (Figure 6(e)) levels compared to the corresponding levels in the absence of NK-2R antagonist treatment.

## 4. Discussion

Previously, we have shown that SP acting via NK-1R was responsible for the leukocyte responses, inflammatory processes, and pulmonary damage in sepsis, and blocking of NK-1R was beneficial to the mice in managing the lung injury in polymicrobial sepsis [3]. We observed that treatment with SR140333, 30 minutes before or 1 hour after CLP, significantly reduced the lung neutrophil infiltration and damage and levels of chemokines, cytokines, and adhesion molecules 8 hours after CLP [3]. So, our next goal was to explore the underlying mechanisms for these beneficial effects of NK-1R antagonism in sepsis. It was important to see how the downstream intracellular signaling was propagated and conveyed to the nucleus. We injected SR140333 either 30 minutes before or 1 hour after CLP, and the protective effect was analysed 8 hours after CLP.

 SP bound to NK-1R is known to upregulate pro-inflammatory cytokines [23]. Activation of inflammatory mediators in sepsis depends mainly on the activation of transcription factor NF-*κ*B [5]. NF-*κ*B is activated by bacterial lipopolysaccharide, cytokines, viral infection, and lung injury. Furthermore, lung epithelial cells have been reported to express cytokine genes in response to injury [24, 25], and NF-*κ*B was activated *in vitro* in these cells by SP [23]. Consistently, our *in vivo* data show that treatment of septic mice with NK-1R antagonist reduced I*κ*B degradation and nuclear NF-*κ*B activity. Although NF-*κ*B inhibition has been reported to improve survival in endotoxin models, the situation is not that straight forward in CLP-induced sepsis [5]. Impaired survival has been reported when NF-*κ*B was inhibited by PDTC [26]. While inhibition of NF-*κ*B decreases the inflammatory mediators, complete loss of antiapoptotic actions of NF-*κ*B might be detrimental in the host defense [5]. We found a lowering of NF-*κ*B activation, but the levels were still elevated compared to the basal levels. In addition, SR140333 treatment in sepsis lowered the activity of another transcription factor, AP-1, which regulates various cytokine and chemokine genes [27]. Thus, SR140333 appears to modulate inflammatory mediators by regulating the activation of NF-*κ*B and AP-1.

 MAPKs signaling cascade is known to activate NF-*κ*B [8, 28, 29]. *In vitro* treatment of Tacr1-expressing cells with SP increased phosphorylation of ERK1/2 MAPK [23]. In addition, SP-NK-1R-NF-*κ*B pathway upregulates pro-inflammatory cytokines in human colonic epithelial cells (via PKC*δ*) [30, 31], monocytes (via ERK) [32], murine macrophages and dendritic cells (calcium independent) [33], human mast cells (via PI3 kinase, PKA) [34], rat peritoneal mast cells (via MAPKs) [35], human T lymphocytes [36], human embryonic kidney cells (via PKC*δ*, ERK) [37], and human mesenteric preadipocytes [38]. We investigated the potential involvement of ERK in mediating SP-NK-1R-NF-*κ*B activation in sepsis. ERK phosphorylation was significantly increased in sepsis, implying a role of ERK in NF-*κ*B activation. However, SR140333 did not reduce ERK level significantly. Tachykinins activate NF-*κ*B by multiple mechanisms involving phospholipase C, calcium, PKC, Ras/Raf/ERK, MAPK/ERK kinase, and I*κ*B degradation [23]. The signaling pathways utilized by SP in cells might vary with multiple G-protein types, the host cell, and signaling components [33]. In colonic epithelial cells, SP-induced NF-*κ*B activation was dependent on PKC*δ* activity, but not calcium or ERK [30, 31]. Furthermore, two isoforms of NK-1R with different binding and signaling properties were identified recently [32]. Thus, possibly other mechanisms might be involved in the SP-NK-1R-mediated signaling in sepsis in addition to ERK.

 NK-1R is a GPCR [23, 39], and activation of Gq, the main G protein associated with NK-1R, is known to stimulate phospholipase C, release intracellular calcium, and activate PKC. A PKC inhibitor is shown to block SP-induced activation of NF-*κ*B *in vitro* [23]. We observed a significant phosphorylation and activation of PKC*α*, 8 hours after sepsis, and SR140333 significantly reduced lung PKC*α* levels. Thus, it is possible to conclude that SP-NK-1R promotes inflammation in polymicrobial sepsis mediated by PKC*α*.

 Expression of NK-1R is reported to be upregulated by noxious stimuli in inflammatory conditions [23, 40, 41]. Increased expression of NK-1R [42] and NK-2R [43] mRNA has been reported in asthmatic airways. As expected, expression of NK-1R and NK-2R in our study was elevated 8 hours after CLP-induced sepsis. Treatment with NK-1R antagonist had no significant change in the receptor expression, although it reduced the lung inflammation in sepsis. 

 Consistent with our earlier study [2], we observed elevated lung and plasma SP levels in sepsis in the absence of SR140333. Although significant, compared to sham the increase was small in magnitude in plasma (more than 1.5 times), but much higher in lungs (more than 3.5 times). Increase in SP levels possibly leads to neurogenic inflammation and pulmonary damage. Blocking the actions of SP did not affect the lung levels of SP. However, it is intriguing that plasma SP levels were lowered by SR140333. It is possible that blocking of SP actions resulted in its increased clearance from the bloodstream, but the local levels at the site of injury remained elevated.

 SP binds NK-1R with high affinity compared to its low affinity to the other tachykinin receptors, NK-2R and NK-3R [44]. NK-2R mRNA, but not NK-3R, has been detected in normal lungs [45]. NK-2R stimulation has been demonstrated to play a role in bronchoconstriction [46, 47], and a selective NK-2R inhibitor has been reported to inhibit bronchoconstriction in asthmatics [48] and to be beneficial in airway disease [49]. We used GR159897, a selective and long-acting NK-2R antagonist [18, 19], to further evaluate if SP mediated its proinflammatory activity in sepsis via NK-2R, in addition to NK-1R. Administration of GR159897 1 hour after CLP failed to reduce MPO levels significantly in septic mice. Also, the chemokines, CCL-2, CXCL-2, and cytokines, IL-1*β* and IL-6, were not affected by NK-2R blocking in sepsis. Tachykinins contract smooth muscles mainly by interaction with NK-2R, while the vascular and proinflammatory effects are mediated by NK-1R [47]. In the absence of GR159897, vehicle-treated mice showed signs of polymicrobial sepsis with elevated MPO activity and lung chemokine and cytokine levels. Thus, it seems probable that proinflammatory activity of SP in polymicrobial sepsis is mediated mainly by NK-1R.

 In conclusion, the present data reveals the possibility that SP acting via NK-1R initiates signaling cascade that is mediated by PKC*α* and leads to NF-*κ*B and AP-1 activation and further modulates proinflammatory mediators in polymicrobial sepsis, and the effect of SP is blocked 8 hours after CLP by NK-1R antagonist SR140333 administered 30 minutes before and 1 hour after CLP. Although earlier work underlined the importance of SP in lung injury associated with polymicrobial sepsis, the new information provided here emphasizes the possible mediators and mechanisms of the beneficial effects of blocking SP receptors. These molecular insights move us closer to understanding and treating sepsis better.

## Figures and Tables

**Table 1:** Primer sequences and optimal conditions used in PCR analysis.

Gene name	Sense primer sequence (5′–3′)	Antisense primer sequence (5′–3′)	Amplification conditions	No. of amplification cycles
NK-1R	CTT GCC TTT TGG AAC CGT GTG	CAC TGT CCT CAT TCTCTT GTGGG	95°C 30 s; 59°C 30 s; 72°C 30 s	38
NK-2R	TGC TGT CAT CTG GCT GGT AG	TCT TCC TCG GTT GGT GTC CC	95°C 30 s; 61°C 30 s; 72°C 30 s	42
18S	GTA ACC CGT TGA ACC CCA TT	CCA TCC AAT CGG TAG TAG CG	95°C 30 s; 59°C 30 s; 72°C 30 s	24

## Abbreviations

AP-1: Activator protein-1
CLP: Cecal ligation and puncture
ERK: Extracellular signal regulated kinase
GPCR: G protein-coupled receptor
HPRT: Hypoxanthine guanine phosphoribosyl transferase
HRP: Horseradish peroxidase
MAPKs: Mitogen-activated protein kinases
MPO: Myeloperoxidase
NK-R: Neurokinin receptor
PBST: Phosphate buffered saline Tween 20
PKC: Protein kinase C
*PPTA:*: *Preprotachykinin-A*
s.c.: Subcutaneous
SIRS: Systemic inflammatory response syndrome
SP: Substance P.
